# Expression and Functional Analyses of the *WIP* Gene Family in Arabidopsis

**DOI:** 10.3390/plants11152010

**Published:** 2022-08-01

**Authors:** David Diaz-Ramirez, Ury Sarai Diaz-Garcia, Guadalupe Magdaleno-Garcia, Gunnar Huep, Ingo Appelhagen, Martin Sagasser, Nayelli Marsch-Martinez

**Affiliations:** 1 Department of Biotechnology and Biochemistry, Center for Research and Advanced Studies of the National Polytechnic Institute, Irapuato 36824, Mexico; david.diaz@cinvestav.mx (D.D.-R.); ury.diaz@cinvestav.mx (U.S.D.-G.); lupita.magdaleno@hotmail.com (G.M.-G.); 2 Genetics and Genomics of Plants, Faculty of Biology, Bielefeld University, Universitätsstraße 27, 33615 Bielefeld, Germany; ghuep@cebitec.uni-bielefeld.de (G.H.); appelha@cebitec.uni-bielefeld.de (I.A.); sagasser@cebitec.uni-bielefeld.de (M.S.)

**Keywords:** Arabidopsis, transcription factor, no transmitting tract, *WIP* gene family, redundancy, organ size, cell size, plant vasculature

## Abstract

The WIP family of transcription factors comprises the A1d subgroup of C2H2 zinc finger proteins. This family has six members in *Arabidopsis thaliana* and most of the known functions have been described by analyzing single knockout mutants. However, it has been shown that *WIP2* and its closest paralogs *WIP4* and *WIP5* have a redundant and essential function in root meristems. It is likely that these and other *WIP* genes perform more, still unknown, functions. To obtain hints about these other functions, the expression of the six *WIP* genes was explored. Moreover, phenotypic ana-lyses of overexpressors and *wip* mutants revealed functions in modulating organ and cell size, stomatal density, and vasculature development.

## 1. Introduction

WIP proteins are plant transcription factors that contain four zinc finger domains. They show nuclear localization and belong to the A1d subgroup of C2H2 zinc finger proteins [1,2]. *WIP* genes have been found in all available plant genomes, and mutants have been studied in *Arabidopsis thaliana*, *Cucumis melo*, *Gerbera hybrida*, and the ancestral plant *Marchantia polymorpha* [1,3,4,5,6]. *Marchantia polymorpha*, a nonvascular plant, has a single *MpWIP* representative in its genome. In this plant, MpWIP is required for the development of the air pore complex, a multicellular gas exchange structure [6]. In melon, it has been reported that the expression of the *CmWIP1* gene leads to carpel abortion, producing male flowers, while plants that produce only female or hermaphroditic flowers are the result of epigenetic modifications in the promoter of the *CmWIP1* gene [4].

Arabidopsis has six members of the *WIP* family (*WIP1* to *6*). Altered phenotypes of single mutants have been characterized and described for only three of them (*wip1*, *wip2*, and *wip6*). In Arabidopsis, the founder member of the family *WIP1/TRANSPARENT TESTA 1* (*WIP1/TT1*) is expressed in endothelial cells during seed development. The loss of its function causes a yellow seed phenotype that contrasts with the wild-type (wt) brown seed. Due to this phenotype, the mutant received the name of *transparent testa 1* (*tt1*). The yellow seed color of these mutants is due to the lack of proanthocyanidins [3]. *WIP6* is also known as *DEFECTIVELY ORGANIZED TRIBUTARIES 5* (*DOT5*) [7]. Loss-of-function *wip6* mutants were reported to present alterations in the venation pattern of the leaves, a delay in leaf and root development, and sometimes alterations in phyllotaxy. It has been suggested that DOT5 is required for normal stem and root development [7].

*WIP2* is the member of the *WIP* gene family that has been most intensively studied, and for which close homologs have been studied in other species. It plays an important role in the development of the transmitting tract, a tissue in the pistil, in which the pollen tubes grow from the stigma to the ovules. The loss of its function affects this tissue and the mutant is, therefore, also referred to as *no transmitting tract* (*ntt*) [8]. Moreover, the *WIP2* promoter is active during the development of the replum and the overexpression of this gene increases its size, while mutants with loss-of-function have the opposite effect with a reduction in the number of cell rows [9]. Its overexpression also affects lignification, causing fruit indehiscence [10]. Protein–protein interaction assays have revealed that WIP2/NTT interacts with different transcription factors [9]. SEEDSTICK (STK) is one of these interactors, and the *stk ntt* double mutant has defective septum fusion [11].

Interestingly, while *WIP2/NTT* is also expressed in more tissues other than the transmitting tract [9,10] including the root meristem [12], the single loss-of-function mutant does not present an evident, severe phenotypic difference to the wild-type. This may be due to redundancy. Indeed, the combination of the mutations in the triple *wip2*/*ntt wip4 wip5* mutant results in plants that do not develop a root [12], revealing an essential, though redundant, function of these genes. This suggests that there are still other unknown functions, and a global exploration of expression and effects of these genes can provide more information about these functions.

In this work, we aimed to explore the function of the members of the Arabidopsis *WIP* family. We analyzed their expression and the effects of overexpression and loss-of-function in Arabidopsis.

## 2. Results

### 2.1. Expression Pattern of the WIP Gene Family

**Figure 1:**
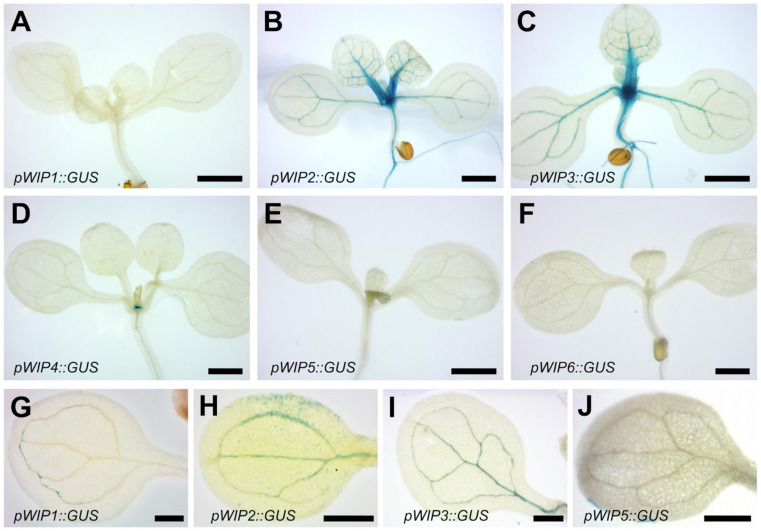
***pWIP::GUS* expression in 9 dag seedlings**. Scale bars represent 1 mm in (**A**–**F**) and 0.5 mm in (**G**–**J**).

To visualize the expression patterns of the six different Arabidopsis *WIP* genes, representative independent GUS reporter lines for each of the promoters of the family members were evaluated (Figure 1). Aerial organs at different developmental stages were analyzed. First, the pattern of GUS expression in seedlings was followed each day, for the first 9 days after germination (dag). Staining in the different lines showed a constant pattern during these first days for the different reporter lines, and Figure 1A–F show stained 9 dag seedlings. Expression was observed in the SAM for *pWIP2*, *pWIP3*, and *pWIP4*, and in the hypocotyl vasculature for *pWIP2* and *pWIP3*. There was also expression of *pWIP2* and *pWIP3* and faint expression in *pWIP1* in the cotyledon vasculature (Figure 1G–I), and expression of *pWIP2* and *pWIP3* in the leaf vasculature (Figure 1B,C). We also observed expression in the root for *pWIP2*, *pWIP3*, *pWIP4*, and *pWIP5.* Figure S1 presents expression of *pWIP2* and *pWIP4* in the main and lateral roots. This coincides with data from publicly available databases (Figure S2). For other tissues, there were also coincidences, especially for those where there is strong GUS staining, though RNA-seq counts were low or very low for those tissues where GUS staining was faint.

**Figure 2:**
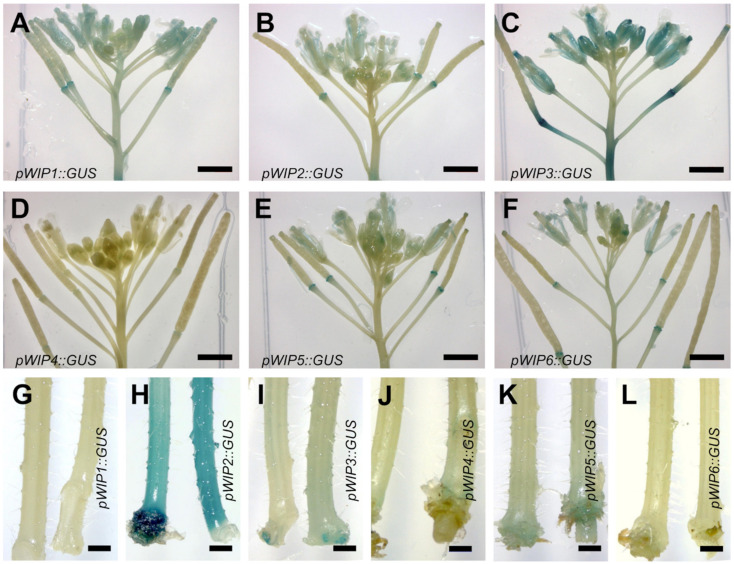
***pWIP::GUS* expression in inflorescences and the base of inflorescence stems**. (**A**–**F**) Inflorescences; (**G**–**L**) base of the inflorescence stem. Scale bars represent 1 mm.

Next, expression in inflorescences was evaluated (Figure 2A–F). This evaluation included the stem (Figure 2G–L). Expression was observed in diverse tissues in the inflorescences. *pWIP1*, *pWIP2*, *pWIP3*, *pWIP5*, and *pWIP6* were expressed in different floral organs, such as petals, sepals, and stamens.

Interestingly, all 6 *WIP* reporter lines showed expression at the top of the pedicels to which developing fruits, or siliques, were attached, and the abscission zone (Figure 2A–F), though it was faint for some lines. For some genes, the lower part of the valves also showed expression. Similarly, the base of the stem of the 6 *WIP* reporter lines showed staining, although it was very faint in *pWIP1* and *pWIP6* (Figure 2G–L). *pWIP2* presented very strong staining in this tissue (Figure 2H). A stem section of the *pWIP2* reporter line is shown in Figure S1, where blue staining is observed in the internal tissues of the stem, associated to the vasculature.

The expression in the analyzed tissues is summarized in Table S1. There were different tissues where the expression of 2 or more *WIP* genes appeared to overlap, for example, in the shoot apical meristem region for *pWIP2*, *pWIP3*, and *pWIP4* or cotyledon vasculature for *pWIP1*, *pWIP2*, and *pWIP3*. A coincidence of expression in young aerial tissues was found for *pWIP2* and *pWIP3*. Finally, staining was observed at the connection of the silique base and the top of the petiole, and the base of inflorescence stems for all family members. This suggests that there might be redundant functions in at least some of these tissues.

### 2.2. Overexpression of the WIP Gene Family Causes Leaf and Cell Size Reduction

**Figure 3:**
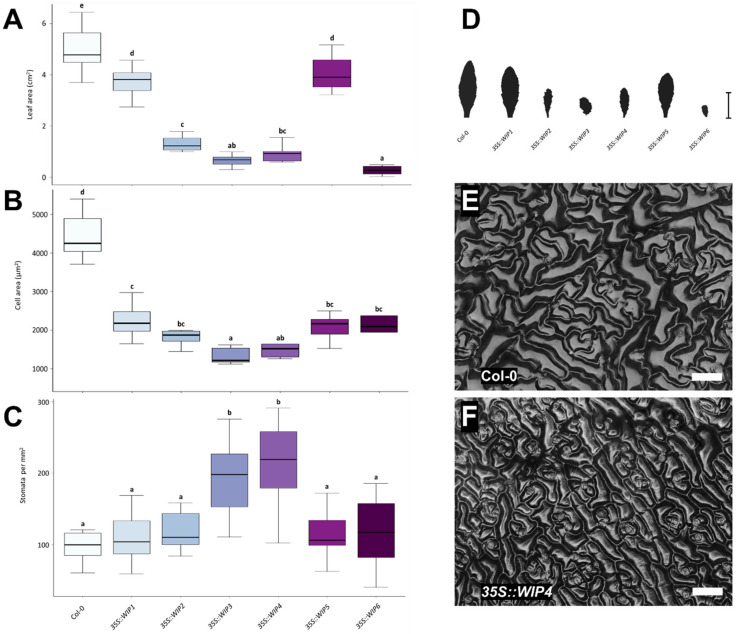
**Leaf phenotypic analyses of *WIP* overexpressors (OE)**. (**A**) Leaf area of *WIP* OE. (**B**) Leaf cell area of *WIP* OE. (**C**) Stomatal density in *WIP* OE leaves. (**D**) Representative leaf shape and average size of *WIP* OE. (**E**) Representative micrograph of leaf pavement cells in wt (Col-0) (**E**) and *35S::WIP4* (**F**) leaves. Scale bars represent 1 cm in (**D**) and 40 µm in (**E**) and (**F**). In (**A**–**C**), ANOVA *p*-value < 0.0001. Letters represent different statistical groups (LSD test).

Next, to further gather information about the function of *WIP* genes as a family, the effects of the overexpression of all 6 family members were evaluated (Figure 3). At first sight, it was evident that rosette leaves were affected in all overexpressors. They were different to wild-type leaves (Figure S3), and their edges showed irregularities such as serrations, undulations, indentations, or lobulations, depending on the gene that was overexpressed. Moreover, leaf size appeared to be affected. To evaluate this in more detail, total leaf area was measured. The leaf area of all overexpressors was smaller than the area of wild-type leaves (Figure 3A,D).

From the overexpressors, *35S::WIP2*, *35S::WIP3*, *35S::WIP4*, and *35S::WIP6* were the smallest, while *35S::WIP1* and *35S::WIP5* had an intermediate total leaf area size. Next, the size of cells (cell area) in these leaves was analyzed, and they were also found to be smaller than wild-type cells (Figure 3B). This was the case for all overexpressors, with *35S::WIP3* and *35S::WIP4* presenting the smallest cells. As an example, Figure 3E,F show the comparison of leaf cells of wild-type plants, and plants overexpressing *WIP4*. While analyzing cell area, some differences were noticed in the spacing between stomata. Therefore, stomata were counted in the imaged area, and the stomatal density was calculated (Figure 3C). Most overexpressors did not show statistically significant differences. However, *35S::WIP3* and *35S::WIP4* leaves had a higher stomatal density than wild-type leaves (Figure 3C), which could indicate that they affect stomata or pavement cell development, which should be further studied.

Moreover, to further explore the possible cause of the size reduction in leaves, the total number of cells in the leaves was estimated using the leaf size and cell size data. Interestingly, the estimated number of cells differed in the overexpressors and wild-type leaves (Figure S4). The estimated number of cells was higher for *WIP1* and *WIP5* overexpressors, and lower for the rest of the *WIP* overexpressors, suggesting that the ectopic and constitutive expression can affect cell proliferation.

In summary, the overexpression of all *WIP* genes caused alterations in leaf shape, leaf size, and cell size, reducing them in comparison to wild-type leaves. It also affected cell numbers. Moreover, the overexpression of *WIP3* and *WIP4* also increased stomatal density.

### 2.3. Loss of WIP Function Affects Leaf and Cell Size

**Figure 4:**
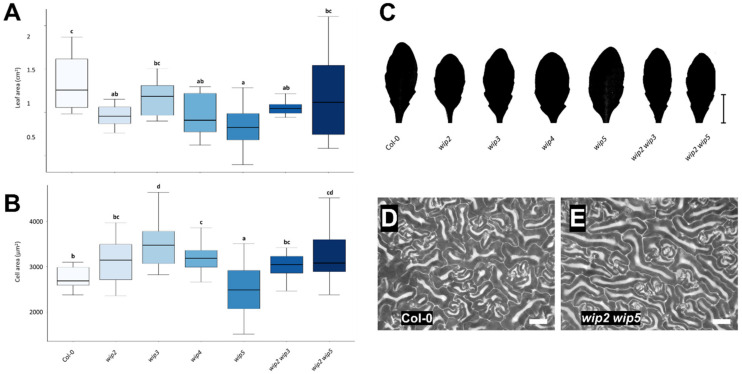
**Leaf phenotypic analyses of *wip* mutants at 30 dag**. (**A**) Leaf area of *wip* mutants. (**B**) Leaf cell area of *wip* mutants. (**C**) Representative leaf shape and average size of ***w****ip* mutants. (**D**) Representative micrograph of leaf pavement cells in wt (Col-0) and (**E**) *wip2 wip5* leaves. Scale bars represent 1 cm in (**C**) and 40 µm in (**D**) and (**E**). ANOVA *p*-value = 0.0113 in (**A**) and 0.0008 in (**B**). Letters represent different statistical groups (LSD test).

**Figure 5:**
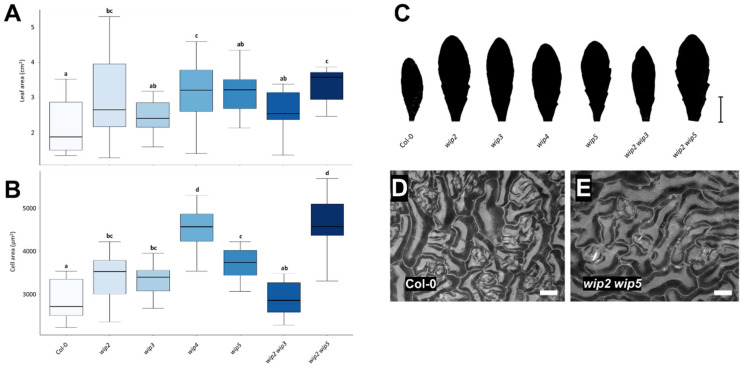
**Leaf phenotypic analyses of *wip* mutants at 60 dag**. (**A**) Leaf area of *wip* mutants. (**B**) Cell area of *wip* mutants. (**C**) Representative leaf shape and average size of *wip* mutants. (**D**) Representative micrograph of leaf pavement cells in wt (Col-0) and (**E**) *wip2 wip5* leaves. Scale bars represent 1 cm in (**C**), and in (**D**) and (**E**) 40 µm. ANOVA *p*-value= 0.0141 in (**A**) and 0.0001. in (**B**). Letters represent different statistical groups (LSD test).

Next, the leaves of *WIP* loss-of-function mutants were analyzed (*wip2*, *wip3*, *wip4*, and *wip5* single, and *wip2 wip3* and *wip2 wip5* double mutants). Figure 4C–E show representative images used for the analyses. Two different time points, 30 and 60 days after germination (dag), were evaluated (Figure 4 and Figure 5).

At 30 days, the leaves of *wip2*, *wip4*, *wip5*, and *wip2 wip3* mutants had a smaller size (Figure 4A,C). The rest of the mutants had leaves that were the same size or slightly smaller than that of the wild-type.

When cell size (cell area) was evaluated, the epidermal cell area in mutant *wip3*, *wip4*, and *wip2 wip5* leaves was larger, while it was very variable in *wip5* leaves (Figure 4B). The cell size of the rest of the *wip* mutants did not differ much from that of the wild-type, though there was a tendency for leaves to have a larger cell size in the *wip2* and *wip2 wip3* mutants (Figure 4B).

As some mutants had smaller leaves and larger cells than wild-type leaves, this could mean that cell number was also affected. The number of cells in the leaf was estimated from the number of cells in the evaluated leaf area. This is only an estimation because the size of the cells would differ in the whole leaf. Nevertheless, in this evaluation, there was a tendency for a reduced number of estimated cells in all the mutants in comparison to wild-type leaves (Figure S4). This reduction in the estimated number of cells per leaf was marked in the single *wip2* and *wip4* mutants (Figure S4).

The second leaf and cell size evaluations were performed using the same plants one month later, at 60 dag (Figure 5). At this time, *wip2*, *wip4*, and *wip2 wip5* had larger leaves than wild-type plants, while *wip3*, *wip5*, and *wip2 wip3* had a slightly larger but, in general, comparable size (Figure 5A,C).

When cell area was evaluated, all analyzed mutants had larger cells than wild-type leaves, except for the double *wip2 wip3* mutant (Figure 5B). Representative images of *wip2 wip5* and wild-type leaf cells are presented in Figure 5D,E, and a SEM micrograph of *wip2 wip5* and wild-type cells where cells can be visualized with more detail is shown in Figure S5.

The number of cells in the leaves was again estimated (Figure S4). At this time, there were no statistically significant differences, though there were some mutant genotypes that appeared to have a tendency for an increased estimated number of cells than wild-type leaves (Figure S4).

Finally, because the overexpression lines of *WIP3* and *WIP4* presented a higher stomatal density than wild-type leaves, the stomatal density of the single *wip3* and *wip4* mutants was also evaluated (Figure S6). As a comparison, the double mutant *wip2 wip5* was also included in this analysis. Interestingly, *wip3* and *wip4* had a lower stomatal density than wild-type leaves, while the stomatal density in *wip2 wip5* was not different compared to in wild-type leaves. This indicates that the loss of *WIP3* and *WIP4* function affects stomatal density.

From these analyses, it appears that the loss-of-function of most *WIP* genes affects leaf and cell size in mature plants. Furthermore, the lack of certain *WIP* genes also alters cell number at an earlier stage. This indicates that *WIP* genes are involved in cell growth and some of them possibly in division processes that finally affect normal organ size. Moreover, *WIP3* and *WIP4* also appear to modulate stomatal density.

### 2.4. Wip Phenotype in Stems

**Figure 6:**
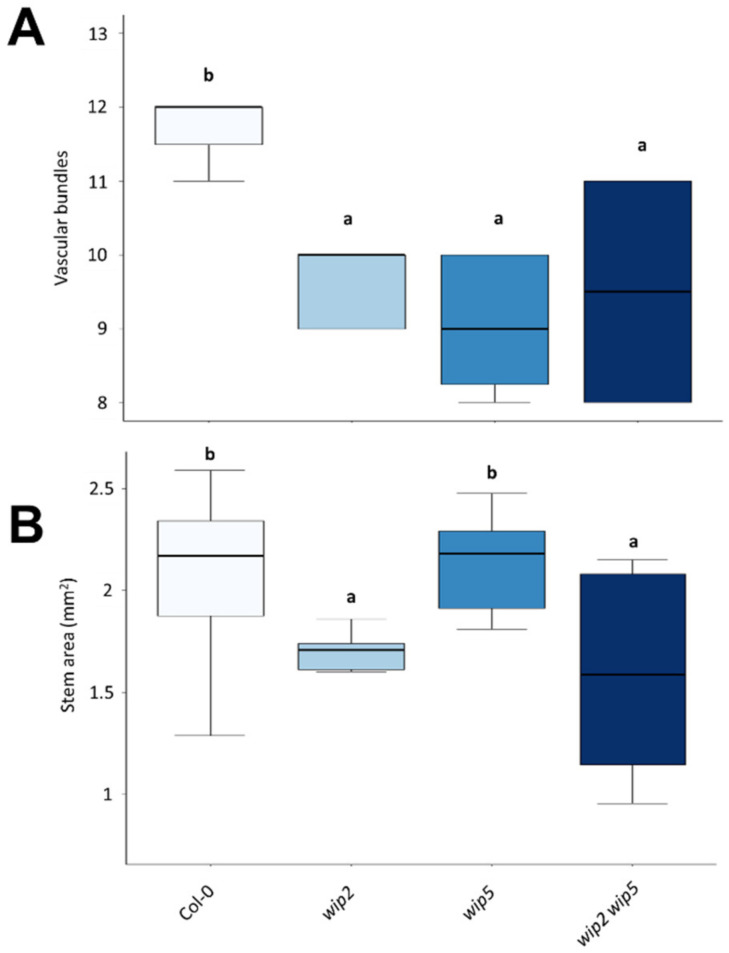
**Stem phenotypic analyses of *wip* mutants.** (**A**) Number of vascular bundles in *wip* mutants. K.W. test *p*-value < 0.0001. (**B**) Stem transverse section area (reflecting stem thickness) of *wip* mutants. ANOVA *p*-value = 0.0013. Letters indicate different statistical groups ((**A**) Dunn’s; (**B**) LSD).

As the expression analyses suggested that *WIP* genes could have a function in the stem and vasculature, the vasculature of mutant stems was compared to wild-type stems. For this, histological sections of 20 cm tall stems of *wip2*, *wip5*, *wip2 wip5*, and wild-type plants were evaluated (Figure 6). They were chosen because GUS staining in the stem was strongest in the *pWIP2* lines, and because some redundancy had been observed in the leaf analyses between *wip2* and *wip5* mutants. Sections obtained 5 cm from the apex were stained with phloroglucinol to visualize lignin (Figure S7). *wip* mutant stems had fewer vascular bundles than wild-type plants (Figure 6A). On the other hand, stem area was different between *wip2* and *wip2 wip5* mutants with respect to wild-type and the *wip5* mutant (Figure 6B). This indicates that these *WIP* genes are involved in the normal development of these tissues.

In summary, the expression of *WIP* genes, the effect of their overexpression and loss-of-function mutation in leaves, and the stem vasculature of two mutants and the combined mutations were analyzed. The conclusion of the obtained data is that *WIP* genes are expressed in different tissues throughout plant development, in some of which they may play a redundant function. Moreover, the results indicate that, depending on the gene, *WIP* genes have an effect on leaf and leaf cell size and number, stem development, and leaf stomatal density at specific stages.

## 3. Discussion

The goal of this work was to obtain insights about the function of the WIP family of transcription factors, by analyzing their expression and the effects of their overexpression and loss-of-function in Arabidopsis rosettes. 

### 3.1. Expression Patterns of WIP Family Promoters

We explored the expression pattern of all six Arabidopsis *WIP* promoters in aerial tissues of seedlings, inflorescences, and stems using transcriptional fusion reporter lines. Interestingly, we observed some tissues where two or more genes were expressed, such as the shoot meristematic region or the vasculature of cotyledons or leaves. Moreover, there were tissues where all genes were expressed, as revealed by GUS staining with different intensities depending on the gene. Interestingly, these tissues are regions where two different organs are connected, such as the pedicel to developing siliques, and the base of the stem that connects to the roots. This suggests that *WIP* genes could have yet undiscovered redundant functions in these and other tissues, particularly the vasculature. These functions may be essential, as it was previously reported for the redundant role of *WIP2*, *WIP4*, and *WIP5* in root development [12].

### 3.2. WIPs Alter Leaf Shape and Size, and Leaf Cell Size

As a strategy to obtain more information about the function of *WIP* genes, the rosette leaf phenotype of overexpressors of the 6 *WIP* genes was evaluated. Interestingly, they all affected leaf size and shape, especially leaf margins. This again suggests that they might perform functions in the regulation of similar processes. In an earlier study [1], the function of *WIP1* could be partially recovered by expressing the other *WIPs* under the *WIP1* promoter. Still, the complementation was not total. Similarly, though all WIPs affect leaf size and edges when overexpressed, the phenotypes are not identical, suggesting the existence of shared as well as specific functions for them. For example, an effect in stomata density was observed, but only in the overexpressors of *WIP3* and *WIP4*. This might be a particular effect that could reflect a specific, different function, and should be studied in more detail in the future.

On the other hand, the effects on organ size and cell size appear to be a function shared by all members of the family. They all affected leaf and cell size, as the analyzed leaves of all overexpressors were smaller and had cells that were also smaller than those of wild-type leaves. Interestingly, the overexpression of a *WIP* gene from *Gerbera hybrida*, *GhWIP2*, with the highest sequence homology to *AtWIP2*, also caused a reduction in organ size [5]. The leaves and floral organs of *GhWIP2* overexpressors were smaller than wild-type organs. Moreover, as noted in this work for Arabidopsis *WIP* overexpressors, cell size was also reduced in *GhWIP2* overexpressor organs. Therefore, as the overexpression of all Arabidopsis *WIP*s has a similar effect on organ and cell size, cell growth modulation might be a common *WIP* function, at least for the six Arabidopsis *WIP*s and Gerbera *GbWIP2*. The same negative effect on cell growth was also reported recently by [13] for the Arabidopsis *WIP* members *WIP1* and *WIP2*. In global expression analyses of overexpression lines, the authors found gene categories related to stress and cell growth repression and they observed a reduction in plant size in these lines [13].

Interestingly, WIPs may have a different effect depending on specific cell types while still affecting cell growth. The overexpression of *WIP2* has the opposite effect in replum cells, as they become very large [9]. This different effect in specific cells or tissues may depend on the presence of other genes, hormones, or factors that modulate WIP activity or effects. For example, in the ovary context, WIP2, together with the transcription factor STK, delays entry of septum cells to their normal degradation program [11].

Finally, the estimated number of cells in the leaves of the overexpressors of *WIP* genes is different (higher or lower) from wild-type leaves (Figure S4A), suggesting that these genes can affect cell proliferation, most likely also depending on the context. In the replum, the overexpression of *WIP2*, in addition to causing a clear enlargement of cell size, reduces the number of cells in this region [9]. This could be due to the presence of other transcription factors or hormonal contexts that modulate the activity and effect of WIP transcription factors.

### 3.3. Loss of WIP Function Affects Leaf and Cell Size

After analyzing the effect of the overexpression of *WIP* genes, the effect of their loss-of-function was evaluated. The genotypes included for this evaluation were *wip2*, *wip3*, *wip4*, *wip5*, *wip2 wip3*, and *wip2 wip5*. Notably, when the phenotype of *wip* single and double loss-of-function mutants was analyzed, some of them presented differences in leaf size that were opposite to the phenotypes presented by the overexpressors.

The results of the analyses of leaves at 30 and 60 days suggest that *WIPs* affect leaf cell division and growth. At 30 days, four *wip* mutants had smaller leaves but a similar cell size, suggesting that these leaves had fewer cells. The single mutants with small leaves were *wip2*, *wip4*, and *wip5*. Interestingly, the single mutant *wip3* had larger cells but a wild-type leaf size. These leaf and cell size relations suggested that these leaves contained fewer cells. When the cell number was estimated, there was indeed a tendency for having a reduced estimated number of cells. This could mean that these *WIP* genes affect processes such as cell division and growth. At 30 days, the loss of *WIP3* function had the strongest effect on cell expansion among all the mutants. The effect in cell division and growth might be a nonautonomous effect, as some of these *WIPs* were expressed at the SAM and leaf or cotyledon vasculature, but not in the whole leaf lamina.

Interestingly, at 60 days, the *wip2*, *wip4*, and *wip2 wip5* mutants had larger cells than wild-type plants, and the rest had wild-type-sized leaves. All evaluated mutants had larger cells, except *wip2 wip3* that had a comparable size to wild-type cells. When cell numbers were calculated, they were not statistically significantly different to the estimated cell numbers in wild-type leaves. Possible explanations for the estimated leaf cell numbers at 60 days could be that cell division was not affected in the analyzed leaves or that cell division continued in the cells that were analyzed at 60 days, reaching a similar number. Nevertheless, it would be very interesting to analyze cell division processes in detail in this or other organs.

In summary, the processes of cell division and growth in leaves at 30 days and cell growth in leaves of mature plants appear to be affected by the lack of *WIP* genes, especially *WIP4* and the combination of *WIP2* and *WIP5*, most likely in a nonautonomous fashion. These three genes are the most closely related and have been found to perform redundant functions in the root [12].

The relation of total leaf area and cell size and estimated number at 30 days resulting from the lack of *WIP* function could also mean that a compensation or overcompensation mechanism is activated in some of the mutants, in which cell size increases to compensate for a smaller number of cells or vice versa [14]. The observation that increased expression of *WIP* genes caused a reduction in cell size in leaves, while the loss of their function caused an increase in cell size in mature plant leaves, suggests that they negatively influence leaf cell size. In some mutants, leaf cell numbers were also altered, so the affected genes might also play a role in cell division, and maybe the timing, duration, or window of active cell division. Interestingly, estimated cell numbers were also affected in plants that overexpress *WIP* genes. This indicates that imbalances in the expression of these genes, either too much or too little expression, can affect cell proliferation. Most likely, this effect is different depending on the context (tissue or developmental stage).

Another interesting observation was the increased stomatal density in the *35S::WIP3* and *35S::WIP4* lines, which contrasted with the decreased stomatal density in the *wip3* and *wip4* mutants. These effects suggest that *WIP3* and *WIP4* may also play a role in normal stomatal density and should be further studied. Regarding possible redundancy, *wip2* and *wip5* appear to share some redundant functions in leaf development, as observed in mature 60-day-old plants, because the phenotypes were stronger in the double *wip2 wip5* mutant than in the single mutants at this stage. At 30 days, this was not the case. Moreover, *wip2* and *wip3* seemed to counteract each other, as the double *wip2 wip3* mutant appeared to have a milder phenotype than the most affected *wip2* or *wip3* single mutants. Furthermore, the effect of the mutations in the size of leaves and cells was different depending on the age of the plant at which the leaf phenotype was analyzed. It would be interesting to evaluate whether environmental conditions can also affect the influence of these mutations on the leaf and other organ’s size and number. Moreover, it would be very informative to further analyze the nonautonomous effect of these genes in leaves and other organs and tissues, by exploring possible mobile signals.

Very recently, the role of the *WIP* family genes in early root development was reported [15]. Interestingly, even when this work was performed using very young roots, some of the observations are in accordance with those of the work presented here, such as nonautonomous effects, and aspects of their expression (such as *pWIP3* and *pWIP6* in siliques). Moreover, the authors report that the combination of *wip1 wip3* and *wip6* mutations recovers the affected root phenotype of the triple *nww* (*ntt wip4 wip5*) mutant, indicating both redundant and opposing roles among the family members [15]. Interestingly, the authors found that, for the case of the developing root, the opposite roles are related to the different expression pattern of the genes, and not to the intrinsic activity of the protein [15]. It would be very interesting to explore if this is also the case for the effects observed in the present work. Finally, the vasculature phenotype of the double *wip2 wip5* mutant suggests that these genes may play a redundant role in vasculature development. It is highly likely that *wip4* also plays a redundant role in this tissue, considering the redundant roles they play in embryo development [12]. Considering the observation of the opposing effect of *wip3* in this work, and of *WIP1*, *WIP3*, and *WIP6* in [15], it is possible that these three genes could have an opposite effect. These functions should be studied in more detail to understand the roles that these genes play in vasculature development.

## 4. Materials and Methods

### 4.1. Plant Material and Constructs

The *TT1/WIP1 promoter::GUS* line was described in [3] and the *NTT/WIP2 promoter::GUS* line was described in [11]. *Promoter::GUS* lines for *WIP3-6* were prepared accordingly using the primers and restriction endonucleases listed in Table S2. The promoter::GUS constructs were transformed into *A. thaliana* plants of the ecotype Columbia, transgenic plants were selected, and corresponding T2 plants were subjected to GUS staining. For each *pWIP* construct, 3 to 6 independent T2 lines were selected and 10 to 20 individuals from each line were stained for the different developmental stages. Whole seedlings 5 and 11 days after germination, inflorescences, stems of adult plants, and immature seeds were analyzed. Expression that occurred consistently in all independent lines was assumed to be construct-specific. In most cases, however, the GUS staining differed only in intensity. For the analyses presented in this work, 2 independent lines that showed the representative staining pattern were used for the assays and 1 was selected for the detailed observations.

Construction of the *35S::TT1/WIP1* and *WIP2*, *3*, *4*, and *6* fusions was described in [1]. *35S::WIP5* was prepared accordingly using the primers listed in Table S2. The resulting *35::WIP* cassettes were subcloned via *Cla*I/*Sac*I into the binary vector pGPTV [16] in the case of *WIP2*, *WIP4*, *WIP5*, and *WIP6*. For *WIP3*, *Nco*I and *Asp*718 were used to subclone the CDS behind the *35S* promoter in pJAN (a derivative of pPAM (GenBank AY027531)). *A. thaliana* Col-0 was transformed by floral dip [17]. For the transgenic overexpression plants (*35S::WIP* cDNA constructs), the presence of the transgene in T1 plants was additionally confirmed by polymerase chain reaction. The T2 seeds resulting from the selection were used for phenotypic analysis of the plants The mutant lines that were used in this study are indicated in Table S3 and the primers used for their molecular confirmation are listed in Table S2.

### 4.2. Growth Conditions

Plants were grown under greenhouse or growth chamber conditions (22 °C, 16 h of light) in soil or in vitro, in ½ MS medium supplemented with 0.5% sugar and 1% agar. All lines were in Columbia (Col-0) background.

### 4.3. Leaf and Cell Analyses

The analyses were carried at one time point for overexpressors, and at two time points for loss-of-function mutants, using the same plants for both time points. First, rosette leaves were numbered (Figure S8, the leaf selected was the same leaf number in all the samples). For 30 dag, leaf number 8 was used, and for 60 dag, leaf number 10 was used. Leaves were cut and placed fresh in the KEYENCE VHX 5000 digital microscope, Keyence, Osaka, Japan [18]. Two micrographs were obtained, one from the right side and another from the left side of the midvein of the leaf for each genotype, and 10 leaves per genotype were sampled (Figure S8). Leaf epidermal cell area (i.e., cell size) was obtained using ImageJ software (http://imagej.nih.gov/ij/) (accessed on 02 June, 2022) [19]. For this, the number of cells in the micrograph was counted and divided by the total area of the micrograph. The electron microscope micrographs shown in Figure S5 were obtained using a Zeiss EVO 40 SEM microscope, Carl Zeiss, Oberkochen, Germany.

### 4.4. Expression Analyses

Samples were collected and left in GUS staining solution (0.5% triton, 2.5 mM potassium ferricyanide, 2.5 mM potassium ferrocyanide, 1 M EDTA, and 2 mM X-Gluc in 50 mM NaPO4) at 37 °C overnight. Afterward, plants were cleared in 70% ethanol to remove chlorophyll and distinguish the stained regions, and then observed in a Zeiss stereo microscope.

In silico expression analyses were conducted using the publicly available Arabidopsis RNA-seq library database (Figure S2) [20]. http://ipf.sustech.edu.cn/pub/athrna/ (accessed on 13 July 2022).

### 4.5. Statistical Analyses

Statistical analyses and graphs were produced in RStudio (Version 4.2.0, RStudio team, Boston, MA, USA) [21]. ANOVA was applied to parametric variables, and a Kruskal–Wallis test was applied in the case of nonparametric variables; LSD and Dunn’s post hoc tests were applied when significant.

## 5. Conclusions

In conclusion, the family of *WIP* genes modulates leaf growth and affects cell expansion and division processes. Some of these genes also participate in vascular development and stomatal density modulation. *WIP2* and *WIP5* perform redundant functions, at least at specific stages, and *WIP2* and *WIP3* perform opposite functions in at least some tissues. Finally, it is likely that other functions, some of them crucial, are masked by redundancy.

## Data Availability

Not applicable.

## Supplementary Materials

The following supporting information can be downloaded at: https://www.mdpi.com/article/10.3390/plants11152010/s1, Figure S1: pWIP::GUS expression in roots and stem; Figure S2: RNA expression levels of *WIP* genes in different tissues [20]; Figure S3: Leaf edge phenotypes of *WIP* overexpressors; Figure S4: Estimated cell number in wip mutants; Figure S5: Representative SEM micrographs of leaf pavement cells; Figure S6: Stomatal density in *wip3*, *wip4* and *wip2* *wip5* mutants. Figure S7: Representative image of stem transverse sections; Figure S8: Leaf imaging experiments workflow. Table S1: *pWIP::* GUS expression; Table S2: Primers used for GUS and OE constructs and mutant analyses; Table S3: Mutant *wip* lines used in this study [22,23,24]. References [21,22,23,24] are cited in Supplementary Materials.

## References

[1] Appelhagen I., Huep G., Lu G.H., Strompen G., Weisshaar B., Sagasser M. (2010). Weird fingers: Functional analysis of WIP domain proteins. FEBS Lett..

[2] Englbrecht C.C., Schoof H., Böhm S. (2004). Conservation, diversification and expansion of C2H2 zinc finger proteins in the *Arabidopsis thaliana* genome. BMC Genom..

[3] Sagasser M., Lu G.H., Hahlbrock K., Weisshaar B. (2002). *A. thaliana* Transparent testa 1 is involved in seed coat development and defines the WIP subfamily of plant zinc finger proteins. Genes Dev..

[4] Martin A., Troadec C., Boualem A., Rajab M., Fernandez R., Morin H., Pitrat M., Dogimont C., Bendahmane A. (2009). A transposon-induced epigenetic change leads to sex determination in melon. Nature.

[5] Ren G., Li L., Huang Y., Wang Y., Zhang W., Zheng R., Zhong C., Wang X. (2018). GhWIP2, a WIP zinc finger protein, suppresses cell expansion in *Gerbera hybrida* by mediating crosstalk between gibberellin, abscisic acid, and auxin. New Phytol..

[6] Jones V.A.S., Dolan L. (2017). MpWIP regulates air pore complex development in the liverwort *Marchantia polymorpha*. Development.

[7] Petricka J.J., Clay N.K., Nelson T.M. (2008). Vein patterning screens and the defectively organized tributaries mutants in *Arabidopsis thaliana*. Plant J..

[8] Crawford B.C.W., Ditta G., Yanofsky M.F. (2007). The *NTT* Gene Is Required for Transmitting-Tract Development in Carpels of *Arabidopsis thaliana*. Curr. Biol..

[9] Marsch-Martínez N., Zúñiga-Mayo M.V., Herrera-Ubaldo H., Ouwerkerk B.P., Pablo-Villa J., Lozano-Sotomayor P., Greco R., Ballester P., Balanzá V., Kuijt J.S. (2014). The NTT transcription factor promotes replum development in Arabidopsis fruits. Plant J..

[10] Chung K.S., Lee J.H., Lee J.S., Ahn J.H. (2013). Fruit indehiscence caused by enhanced expression of NO TRANSMITTING TRACT in *Arabidopsis thaliana*. Mol. Cells.

[11] Herrera-Ubaldo H., Lozano-Sotomayor P., Ezquer I., Di Marzo M., Montes R.A.C., Gómez-Felipe A., Pablo-Villa J., Diaz-Ramirez D., Ballester P., Ferrándiz C. (2019). New roles of NO TRANSMITTING TRACT and SEEDSTICK during medial domain development in Arabidopsis fruits. Development.

[12] Crawford B.C.W., Sewell J., Golembeski G., Roshan C., Long J.A., Yanofsky M.F. (2015). Genetic control of distal stem cell fate within root and embryonic meristems. Science.

[13] Roldan M.V.G., Izhaq F., Verdenaud M., Eleblu J., Haraghi A., Sommard V., Chambrier P., Latrasse D., Jégu T., Benhamed M. (2020). Integrative genome-wide analysis reveals the role of WIP proteins in inhibition of growth and development. Commun. Biol..

[14] Horiguchi G., Tsukaya H. (2011). Organ size regulation in plants: Insights from compensation. Front. Plant Sci..

[15] Du Y., Roldan M.V.G., Haraghi A., Haili N., Izhaq F., Verdenaud M., Boualem A., Bendahmane A. (2022). Spatially expressed *WIP* genes control Arabidopsis embryonic root development. Nat. Plants.

[16] Becker D., Kemper E., Schell J., Masterson R. (1992). New plant binary vectors with selectable markers located proximal to the left T-DNA border. Plant Mol. Biol..

[17] Clough S.J., Bent A.F. (1998). Floral dip: A simplified method for Agrobacterium-mediated transformation of *Arabidopsis thaliana*. Plant J..

[18] Lazcano-Ramírez H.G., Gómez-Felipe A., Díaz-Ramírez D., Durán-Medina Y., Sánchez-Segura L., De Folter S., Marsch-Martínez N. (2018). Non-destructive Plant Morphometric and Color Analyses Using an Optoelectronic 3D Color Microscope. Front. Plant Sci..

[19] Schneider C.A., Rasband W.S., Eliceiri K.W. (2012). NIH Image to ImageJ: 25 years of image analysis. Nat. Methods.

[20] Zhang H., Zhang F., Yu Y., Feng L.I., Jia J., Liu B.O., Li B., Guo H., Zhai J. (2020). A Comprehensive Online Database for Exploring ∼20,000 Public Arabidopsis RNA-Seq Libraries. Mol. Plant.

[21] Rstudio Team (2022). RStudio: Integrated Development for R.

[22] Tissier A.F., Marillonnet S., Klimyuk V., Patel K., Torres M.A., Murphy G., Jones J.D. (1999). Multiple independent defective suppressor-mutator transposon insertions in Arabidopsis: A tool for functional genomics. Plant Cell.

[23] Alonso J.M., Stepanova A.N., Leisse T.J., Kim C.J., Chen H., Shinn P., Stevenson D.K., Zimmerman J., Barajas P., Cheuk R. (2003). Genome-wide insertional mutagenesis of *Arabidopsis thaliana*. Science.

[24] Kleinboelting N., Huep G., Kloetgen A., Viehoever P., Weisshaar B. (2012). GABI-Kat SimpleSearch: New features of the *Arabidopsis thaliana* T-DNA mutant database. Nucleic Acids Res..

