# Supplementary material for: Expression and Functional Analyses of the WIP Gene Family in Arabidopsis
Source: Plants (Basel). 2022 Aug 1;11(15):2010. doi: 10.3390/plants11152010 (PMC9425439; doi:10.3390/plants11152010)
Supplement: Supplementary file 1 [file plants-11-02010-s001.zip › plants-1820288-supplementary.pdf]

Supplementary material

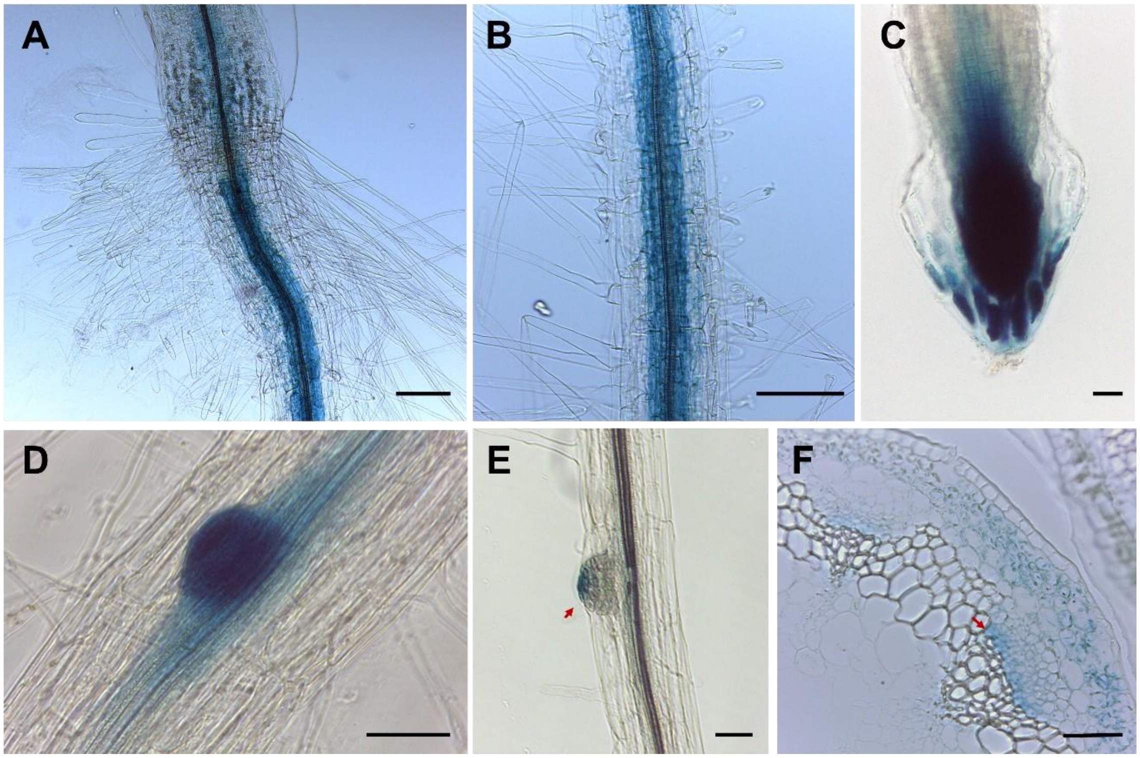

Figure S1. *pWIP::GUS* expression in roots and stem. A) *pWIP2::GUS* expression in the vasculature of the hypocotyl, root and the region between them at 9 dag. B) *pWIP2::GUS* expression in the vasculature of the mature root (9 dag). C) *pWIP2::GUS* expression in the root apical meristem and surrounding tissues. D) *pWIP2::GUS* expression in an emergent lateral root. E) *pWIP4::GUS* expression in the incipient root apical meristem (red arrow) of an emergent lateral root. F) Stem transversal section showing *pWIP2::GUS* expression in vascular bundle (red arrow). Scale bars represent 50 µm.

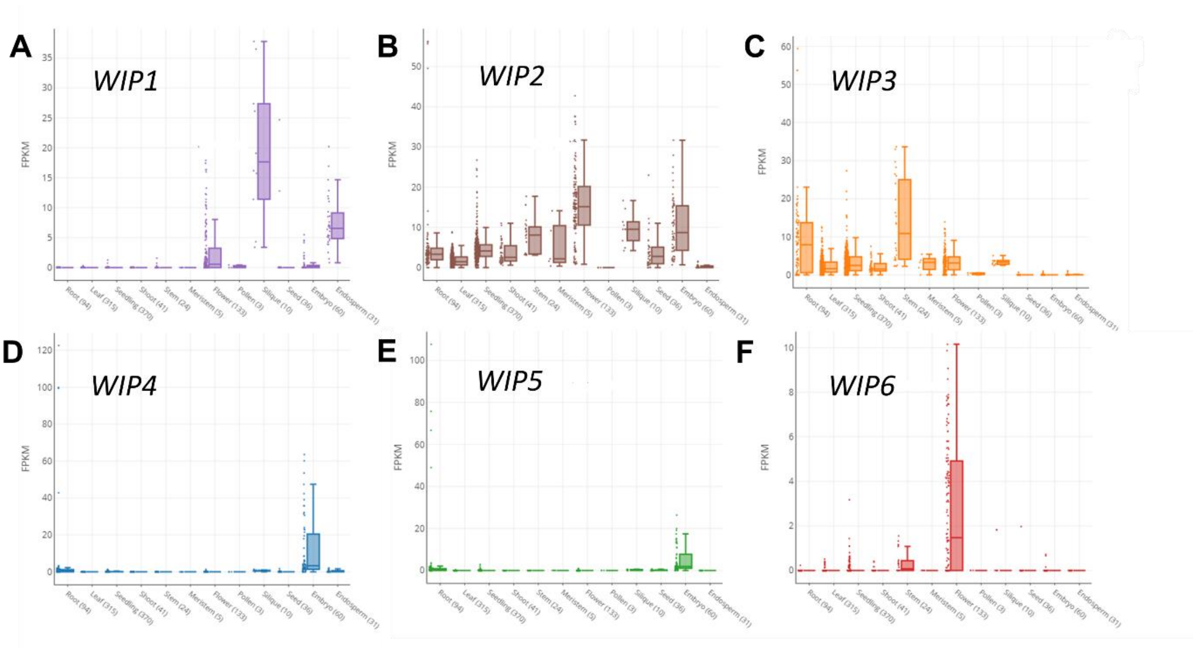

Figure S2. RNA expression levels of *WIP* genes in different tissues. A) AT1G34790 (*WIP1),* B) AT3G57670 (*WIP2*), C) AT1G08290 (*WIP3*), D) AT3G20880 (*WIP4*), E) AT1G51220 (*WIP5*), F) AT1G13290 (*WIP6*). Data obtained from the ARS database [21]

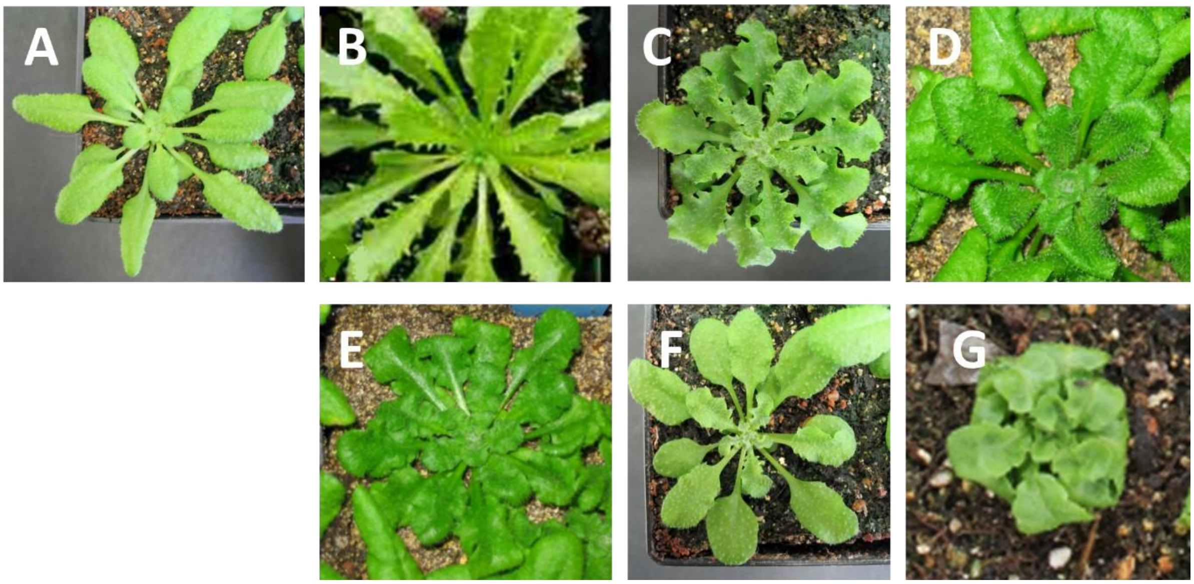

Figure S3. Leaf edge phenotypes of *WIP* overexpressors. A) Col-0, B) *35S::WIP1,* C) *35S::WIP2,* D) *35S::WIP3,* E) *35S::WIP4,* F) 3*5S::WIP5,* G) *35S::WIP6.* Images are presented at different magnifications, to better observe leaf edge alterations, and are not comparable in size.

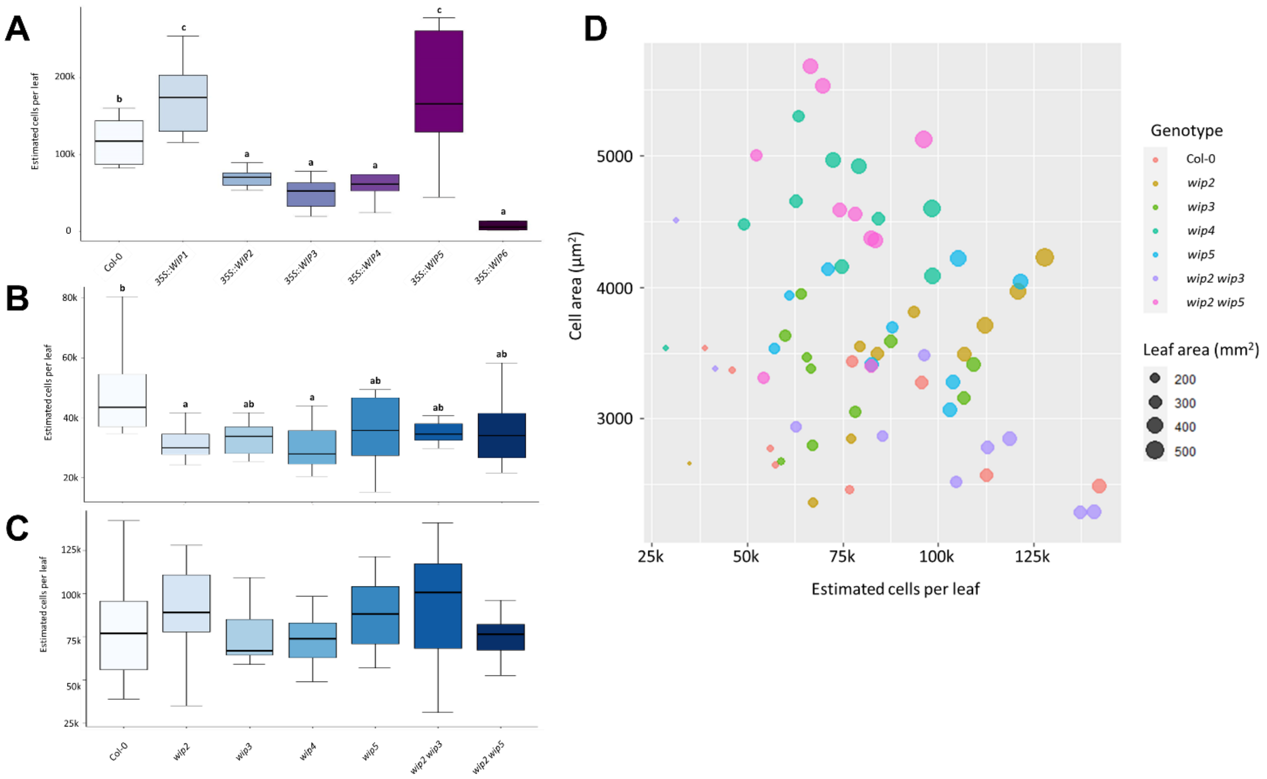

Figure S4. Estimated cell number in *wip* mutants. A) Estimated cell number per leaf at 35 dag in wild type plants and *WIP* overexpressors. B) Estimated cell number per leaf at 30 dag in wild type plants and *wip* mutants. Letters represent different statistic groups. C) Estimated cell number per leaf at 60 dag in wild type plants and *wip* mutants. D) Bubble plot representing the integration of leaf area, leaf cell area and estimated cell number data per leaf, of wild type and *wip* mutant plants at 60 dag.

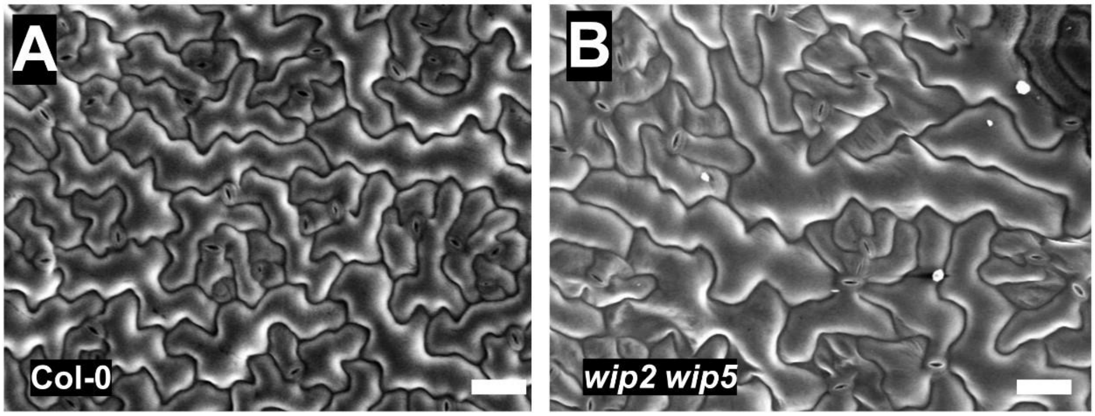

Figure S5. Representative SEM micrographs of leaf pavement cells in wild type (A) and in *wip2 wip5* (B) leaves. Scale bars represent 40 µm.

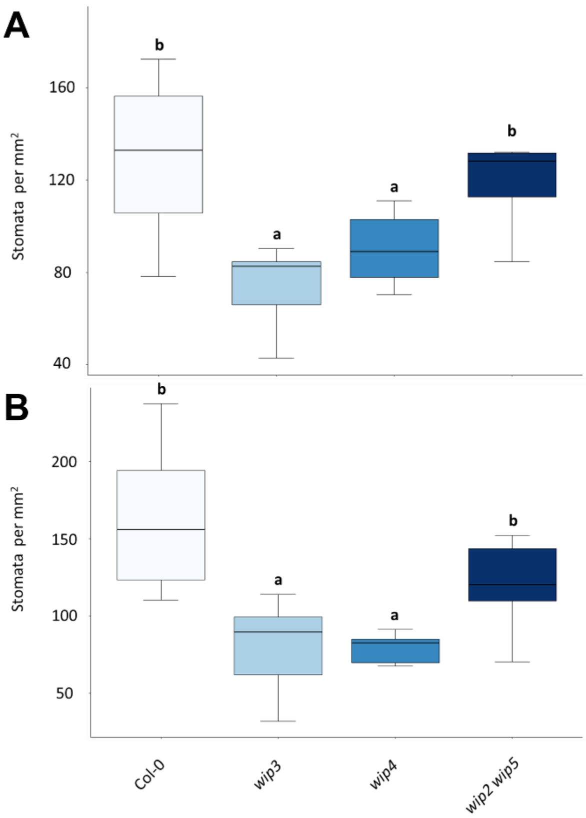

Figure S6. Stomatal density in *wip3, wip4* and *wip2 wip5* mutants. A) Stomatal density of *wip* mutants at 30 dag. B) Stomatal density of *wip* mutants at 60 dag. K.W. test p-value< 0.001 Letter represent different statistical groups. (Dunn's test).

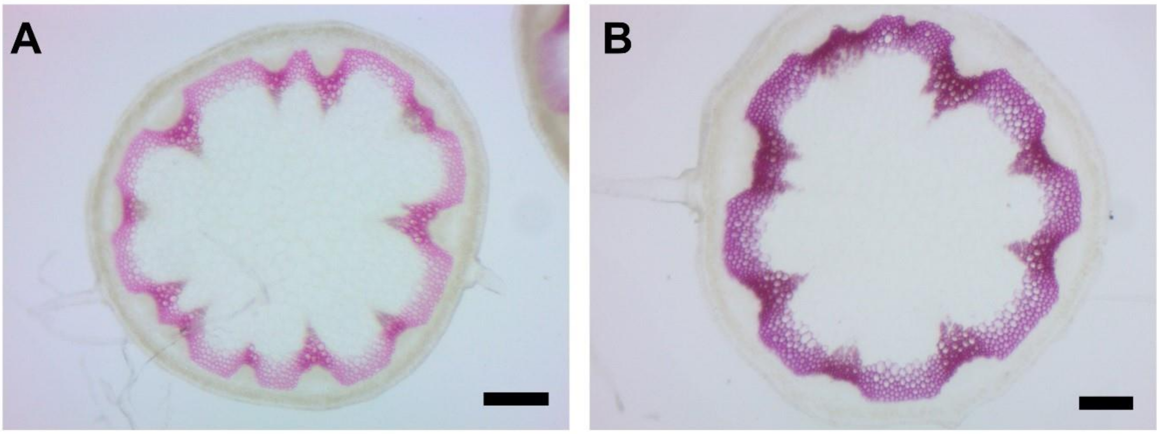

Figure S7. Representative image of stem transverse sections, stained with phloroglucinol, where vascular bundles can be observed. Col-0 (A), and *wip2 wip5* (B) stems. Scale bars represent 200 µm.

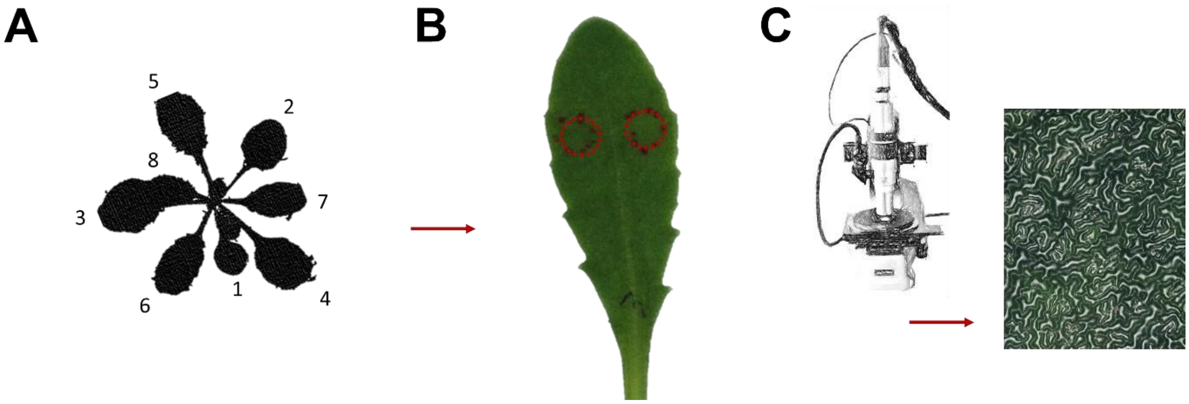

Figure S8. Leaf imaging experiments workflow. A) Leaves were numbered as depicted from older to younger, the 8th leaf was collected at 30 dag and the 10th at 60 dag. B) the same region was micrographed in every leaf, two regions per leaf, in each side of the central vein C) micrographs were taken with a Keyence digital microscope, and images analyzed.

Table S1. ***pWIP::GUS*** **expression.**

Line	SAM	RAM	Silique base	Root vasc.	Cotyledon vasc.	Leaves vasc.	Hypocotyl vasc.	Inflorescence stem vasc.
pWIP1::GUS			✓		✓			✓
pWIP2::GUS	✓	✓	✓	✓	✓	✓	✓	✓
pWIP3::GUS	✓		✓	✓	✓	✓	✓	✓
pWIP4::GUS	✓	✓	✓					✓
pWIP5::GUS		✓	✓					✓
pWIP6::GUS			✓					✓

Table S2. Primers used for GUS and OE constructs and mutant analyses.

Gene Alias	Gene ID	Experiment	Primer Name (site introduced)	Sequence	Fragment length (bp)
WIP3	AT1G08290	promoter	S287 (EcoRI)	TTTGAATTCTAGTACTATTGTATTGG	1528
		GUS line	S288 (NcoI)	TCTCCATGGCTCGCTTTCTCTCCTCTCTTTCAC	
WIP4	AT3G20880	promoter	S311 (EcoRI)	AGTGAATTCATCATGGTAAGCACCTATTAAAGG	1280
		GUS line	S312 (XhoI)	TTACTCGAGAAGATAGTGAGAAAAAAAGAAGAAGAAG	
WIP5	AT1G51220	promoter	S307 (ClaI)	AGAATCGATACGAGACCACCAAAGCAAGTGC	1172
		GUS line	S308 (NcoI)	AGACCATGGCTCTGAGGAGAAGACAAAGAAG	
WIP6	AT1G13290	promoter	S319 (EcoRI)	ATAGAATTCTAATAATATCATCATCACTGAGACTATCAC	1013
		GUS line	S310 (NcoI)	TCTCCATGGAGTAGAGAGAAAGAGAGATGA	
WIP5	AT1G51220	CDS cloning	S245 (NcoI)	AGACCATGGCTTCTAATCCAGCTTGTTCGAATCTC	1014
			S267 (SacI)	TATGAGCTCATTGCTCGATATCAGAAGCAGCATC	
			**Primer name**		**Allele**
WIP2	AT3G57670	Genotyping gene	Sdf222	TCTCTTCACCATGACTGATCC	
			Sdf223	GCTATTTAGACTGCATTGACTC	
		Genotyping insertion	IC77	CTTACCTTTTTTCTTGTAGTG	*ntt-2*
			Sdf277	CATCGTCATCTTCTCCTCCTCC	
			IC51	ATTTTGCCGATTTCGGAAC	*ntt-3*
			Sdf223	GCTATTTAGACTGCATTGACTC	
WIP3	AT1G08290	Genotyping gene	IC13	AGGATGATGTGGTTACCCTCC	
			IC14	TATTGCGAATGATACACCAACCG	
		Genotyping insertion	IC13	AGGATGATGTGGTTACCCTCC	
			IC51	ATTTTGCCGATTTCGGAAC	
WIP4	AT3G20880	Genotyping gene	IC60	GAACTGCGCAAAAATCAAGAG	
			IC61	TCCCTCTCACGCATCTACAAC	
		Genotyping insertion	IC60	GAACTGCGCAAAAATCAAGAG	
			IC62	ATAATAACGCTGCGGACATCTACATTTT	
WIP5	AT1G51220	Genotyping gene	Sdf299	CCTCAGAGTCATGTCTAATCCAG	
			Sdf300	TCTTATTGCTCGATATCAGAAGC	
		Genotyping insertion	IC51	ATTTTGCCGATTTCGGAAC	
			Sdf300	TCTTATTGCTCGATATCAGAAGC	
WIP1	AT1G34790	transcript	S152	ATGGAGTCACCACCACTATAC	
			GH01	AAAAAACAAAGTCTCGGAGAC	
WIP2	AT3G57670	transcript	S185	ACTGATCCTTATTCCAATTTC	
			GH45	ATTTAGACTGCATTGACTCATG	
WIP3	AT1G08290	transcript	S183	ATTCTTATGAGACGAAAGGC	
			GH46	ACTCTGTGGTGACGCATTC	
WIP4	AT3G20880	transcript	S243	CTATTTTCCACTGTTTTGTCACATCG	
			GH47	ATTCTTGCTGCTCGACATCAG	
WIP5	AT1G51220	transcript	S245	TCTAATCCAGCTTGTTCGAATCTC	
			GH48	ATTGCTCGATATCAGAAGCAG	
WIP6	AT1G13290	transcript	S247	TATAATAATAACCAATATAGCTTCTCC	
			GH49	AGTAATTGTCATCAGAAATCTGAC	

Table S3. Mutant *wip* lines used in this study.

wip mutants	Line	Gene ID
wip2 (ntt)	℧SM_3_16705	AT3G57670
wip3	*SALK_072471	AT1G08290
wip4	ϕgabi_176E02	AT3G20880
wip5	*SALK_114838	AT1G51220
wip2wip3	*SALK_007406C,<br>*SALK_072471	AT3G57670,<br>AT1G08290
wip2wip5	℧SM_3_16705,<br>*SALK_114838	AT3G57670,<br>AT1G51220

℧ [22], *[23], ϕ[24].
